# Sirt3 Promoted DNA Damage Repair and Radioresistance Through ATM-Chk2 in Non-small Cell Lung Cancer Cells

**DOI:** 10.7150/jca.53173

**Published:** 2021-07-13

**Authors:** Kun Cao, Yuanyuan Chen, Songyun Zhao, Yijuan Huang, Tingting Liu, Hu Liu, Bailong Li, Jianguo Cui, Jianming Cai, Chong Bai, Yanyong Yang, Fu Gao

**Affiliations:** 1 Department of Radiation Medicine, Faculty of Naval Medicine, Navy Military Medical University; Shanghai, China.; 2 Department of Respiratory and Critical Care Medicine, Changhai Hospital, Navy Military Medical University; Shanghai, China.; 3 School of Public Health & Management Wenzhou Medical University, P.R China.; 4 The First Hospital of Jiaxing, Affiliated Hospital of Jiaxing University

**Keywords:** Sirt3, DNA damage repair, radiosensitivity, lung cancer

## Abstract

**Objective:** Radiotherapy is an indispensable approach for lung cancer, especially for non-small cell lung cancer (NSCLC) with high incidence and mortality. However, cellular resistance to ionizing radiation often results in failure in treatment. In this study, we aimed to investigate the role of Sirt3 in radiotherapy on NSCLC.

**Materials and Methods:** Resected samples from 80 pairs of lung cancer was used to prepare tissue array and Sirt3 was stained with immunochemical method. Cell survival as well as apoptosis assay were used to determine the cellular radiosensitivity. Moreover, DNA damage was evaluated by using γ-H2AX foci. Finally, an *in situ* lung cancer model to test the radiosensitivity in vivo.

**Results:** Sirtuin 3 (Sirt3) was found upregulated in NSCLC cell lines, as well as lung cancer tissues compared with normal tissues. Knockdown of Sirt3 significantly increased radiation-induced cell apoptosis, and increased cell survival efficacy. In contrast, Sirt3 overexpression promoted radioresistance in lung cancer cells. Sirt3 knockdown also aggravated the G2/M cell cycle arrest caused by irradiation. Furthermore, Sirt3 was found to be critical for the activation of ATM-Chk2 pathway upon irradiation. Finally, our in vivo model showed that targeting Sirt3 significantly sensitized lung cancer to radiotherapy.

**Conclusion:** In conclusion, our findings identified a significant role of Sirt3 in radioresistanct of NSCLC, which provides novel mechanism as well as target for radiotherapy.

## Introduction

Non-small cell lung cancer (NSCLC) accounts for approximately 85-90% and remains one of the leading causes of mortality worldwide 1. Besides surgery, chemotherapy as well as targeted therapy, radiotherapy represents a main strategy when used alone or combined with other adjuvant treatment 2. However, the basic or gradually resistance of cancer cells to ionizing radiation often leads to failure of therapy 3,4. The main reason is that the underlying mechanism of radioresistance is unclear and novel radiosensitizing target is to be identified.

Sirtuin 3 (Sirt3) is a member of sirtuins family of NAD-dependent deacetylases which mainly involve in aging and cell fate determination 5. Recently, it has been shown that Sirt3 play critical roles in cancer progression as well as chemoresistance 6,7. Similar to Sirt6 and Sirt7, Sirt3 was also related to DNA damage repair signaling pathway, which is one of core mechanism of cancer radioresistance 8. Previous study showed that Sirt3 is related to the malignancy of NSCLC 9,10. Sirt3 was also found to be involved in cell proliferation, apoptosis and metastasis in lung cancer cells. However, whether Sirt3 plays a role of oncogene or tumor suppressor in different cancer is still controversial. Moreover, there is no evidence whether sirt3 participate in radiation response of NSCLC.

In the present study, we checked the expression of Sirt3 in NSCLC cells and tissues from lung cancer patients. Sirt3 knockdown cells and overexpression cells was generated to study its influence on radiosensivity and the downstream signaling pathways. Furthermore, by using our in situ lung cancer implantation model, we found that Sirt3 deficiency causes increased sensitivity of cancer cells to radiotherapy. Our data provide novel mechanism and target for NSCLC radiotherapy.

## Materials and methods

### Cell culture and lentiviral shRNA infection

Human bronchial epithelial cell line BEAS-2B (American Type Culture Collection, Manassas, VA, USA) and non-small cell lung cancer cell line H1299 (American Type Culture Collection, Manassas, VA, USA) were maintained in RMPI 1640 medium with 10% fetal bovine serum (Gibco, Grand Island, NY, USA), and human non-small cell lung cancer cell line A549 (American Type Culture Collection, Manassas, VA, USA) and mouse lewis lung cancer cell line LLC (American Type Culture Collection, Manassas, VA, USA) were maintained in DMEM medium with 10% fetal bovine serum (Gibco, Grand Island, NY, USA) at 37°C in a 5% CO2 humidified chamber.

Human Sirt3 shRNA (GTGGGTGCTTCAAGTGTTGTT), scrambled shRNA (TTCTCCGAACGTGTCACGT), and overexpresses lentiviral plasmid (Gene sequence from NCBI database) lentiviruses (made from the vector GV248) were generated by BioLink (Shanghai, China). Sirt3 plasmid lentiviralvectors were used for knockdown and overexpression of Sirt3. Cells were seeded at 1×10^5^ cells/well into six-well plates and infected with lentiviral particles using polybrene (10 mg/mL). After infection, virus-containing medium was replaced with normal medium, and then cells were selected with puromycin (2 mg/mL).

### Animals and tumor planting

The whole protocols were approved by the Ethics Committee of Second Military Medical University. Male C57BL/6 mice, 8 weeks old, obtained from the Experimental Animal Center of Chinese Academy of Sciences (Shanghai, China), were used for the animal experiment. Mice were fed in daily-changed individual cages, at 25±1℃ with food and water provided for free access. The mice were randomly divided into three groups: group 1, negative control; group 2, Sirt3 knockdown; group 3, Sirt3 overexpression.

The mouse was anesthetized with isoflurane gas (VETEASY, Shenzhen, China), then placed the right side of the mouse on the operation table. The skin and superficial soft tissue were cut at the midline of left rib, and the insulin injection needle was inserted into the second last rib gap, and the depth of the needle was 2 mm, then the LLC cell was injected into the left lower lobe of the mouse at 1×10^6^ per 25 μl. After skin sutured and disinfected, the mouse was resuscitated.

### Irradiation

The ^60^Co γ-rays in Radiation Center (Faculty of Naval Medicine, Second Military Medical University, Shanghai, China) were applied for the irradiation exposure. Cells were irradiated with 2, 4, 8 Gy at a dose rate of 1 Gy/min. After anesthetization with 10% chloral hydrate (350mg/kg), mice were subjected to whole-lung irradiation with 15Gy at a dose rate of 1Gy/min.

### Western Blot

Cells were homogenized in mammalian protein extraction reagent (M-PER) to prepare a protein sample. The lysates were mixed with 10% SDS-PAGE then electrophoresis was performed on the same amount based on the concentration. After the electrophoresis, the protein was transferred to a nitrocellulose membrane (Amersham, Arlington Heights, IL, USA) then blocked with 5% milk for 1 hr at room temperature. The proteins were incubated with Sirt3 (1:1000), p-ATM (1:1000), p-ATR (1:1000), p-Chk1 (1:1000), p-Chk2 (1:1000), γ-H2AX (1:1000) and β-tublin (1:1000) (Cell Signal Tech, Danvers, MA, USA) at 4°C overnight in a shaker incubator. After washing with TBS-T, the membranes were incubated with anti-rabbit or anti-mouse IgG horseradish peroxidase conjugated antibody (1:5000; Cell Signal Tech.) for 1 hr at room temperature. The protein bands were visualized using enhanced chemiluminescence with a Super Signal west pico kit (Bridgen Biological Technology, Shanghai, China). Films were scanned and analyzed by densitometry using Syngene GeneGenius software (Syngene, Frederick, MD, USA).

### Cell viability assay

Cell viability was determined by Cell Counting Kit-8 (Dojindo, Kumamoto, Japan). Cells were suspended and seeded into 96-well plates with 5×10^3^ cells/well. At the 48h after irradiation, cell viability was tested with a CCK-8 assay.

### Clonogenic assay

Cells were trypsinized, counted, and seeded in 60-mm culture dishes for each dose of radiation. Sufficient numbers were seeded to ensure that approximately 30-100 macroscopic colonies would appear in each plate after 10-14 days. Colonies were stained with 0.5% gentian violet in methanol and counted. The plating efficiency (PE) for each dose was calculated by dividing the number of colonies by the number of cells plated and expressing the result as a percentage. The surviving fraction was calculated by dividing the PE of the irradiation groups by the PE of the appropriate unirradiated control.

### Apoptosis analysis

Apoptosis of cells with or without irradiation was determined by Annexin V-PE and 7-AAD staining. The cells were plated in six-well plates at a density of 10^5^ cells per well and allowed to attach for 24h. The cells were harvested by trypsin digestion, washed with PBS twice, and resuspended 24h after irradiation. Then the cells were stained with Annexin V-PE and 7-AAD at room temperature for 15 min in a dark room according to the Annexin V-PE/7-AAD Apoptosis Detection Kit (YEASEN, Shanghai, China) instructions.

### Cell cycle analysis

Cell cycle was analyzed by PI staining. The cells were plated in six-well plates at a density of 2×10^6^ cells per well and allowed to attach for 24h. The cells were harvested by trypsin digestion in 0, 8, 12, 24h after irradiation, then washed with PBS twice, fixed with pre-cooled 75% ethanol for 24 h, then washed again twice with PBS, and stained with PI for 30 min at 4 ℃, and assayed by flow cytometer.

### Histopathology

At indicated time points, lung tissues were isolated and subjected to sectioning, then the samples were stained with H&E, terminal transferase-mediated dUTP nick end labeling (TUNEL), Ki67 (1:400), Sirt3 (1:200), p-ATM (1:100), p-Chk2 (1:200), and γ-H2AX (1:200) (Cell Signal Tech, Danvers, MA, USA) staining. Five fields per section at 200 magnifications were randomly selected per mouse, and two blinded pathologists independently examined 30 fields per group using Nikon DS-Fi1-U2 microscope (Nikon, Tokyo, Japan).

### Immunofluorescence staining

Immunofluorescence analysis was performed to measure the expression of γ-H2AX in A549 cells. After fixed with 4% formaldehyde solution, the slides were incubated with γ-H2AX (1:200, Cell Signal Tech, Danvers, MA, US) antibodies at 4°C overnight. After washed with PBS, slides were incubated with Texas Red-conjugated anti-rabbit secondary antibodies (Cell Signal Tech, Danvers, MA, US) at room temperature for 30 min. Nuclei were counterstained with DAPI, and the slides were analyzed by using a fluorescence microscope (Nikon Eclipse Ti-SR, Nikon, Tokyo, Japan).

### Statistical analysis

Data was expressed as the means ± standard error of the mean (SEM). Comparison between-group were performed using one‐way ANOVA. Two‐group comparisons were performed using independent‐samples Student's *t*‐test. P<0.05 was considered significant. All experiments were performed at least 3 independent times.

## Results

### Sirt3 is upregulated in NSCLC and responsive to ionizing radiation

**Figure 1:**
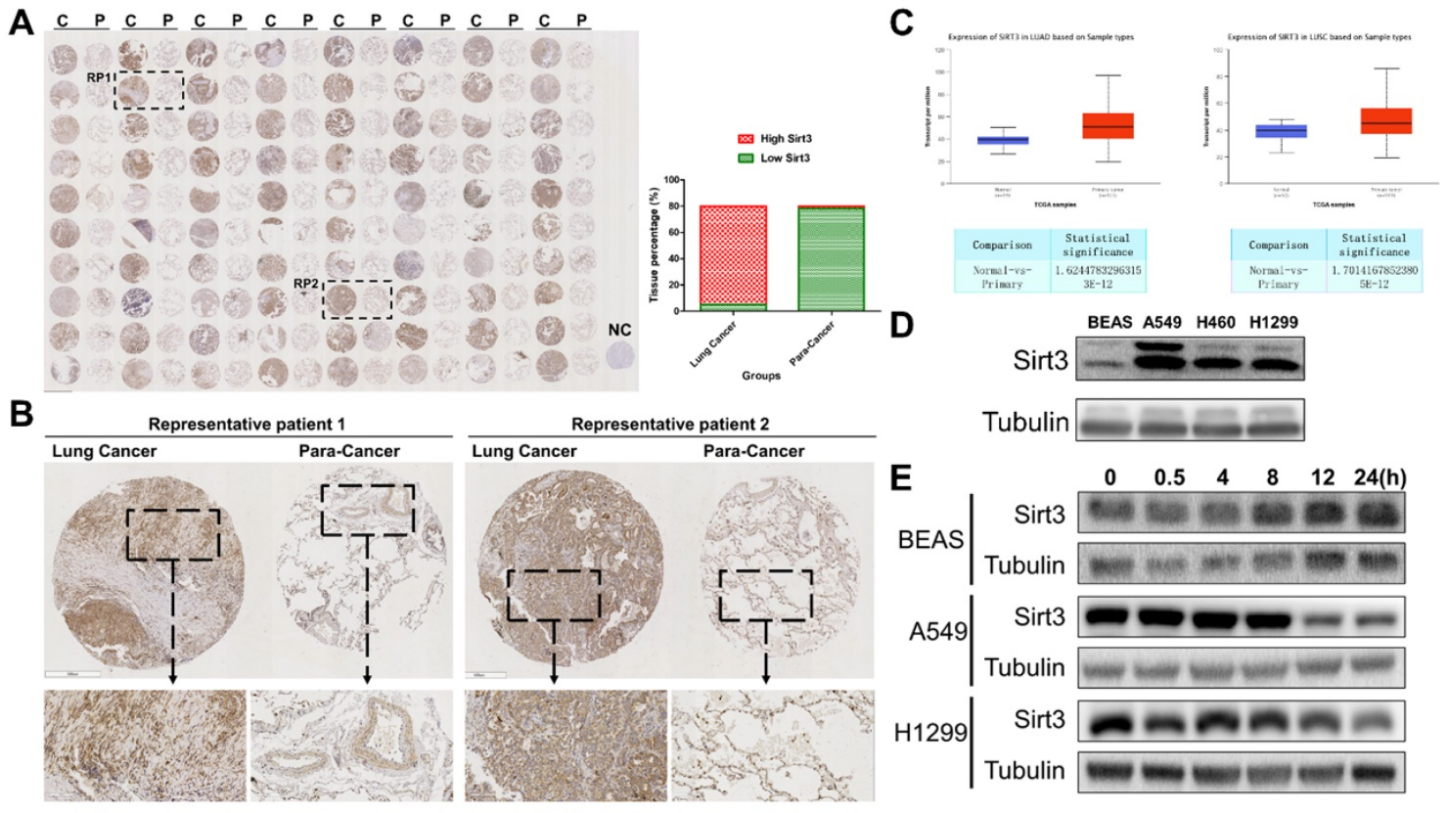
** Sirt3 is upregulated in NSCLC and responsive to ionizing radiation.** (A) Tissue microarray results of Sirt3 expression in clinical lung cancer and their para-cancerous tissue samples. C, P, and NC represent clinical lung cancer tissue samples, para-cancerous tissue samples, negative control, respectively. RP represent the samples of representative patients in Fig 1B. n=80. (B) Immunohistochemical staining results of sirt3 of lung cancer and para-cancer tissues in representative patients. (C) The expression of Sirt3 in lung adenocarcinoma (n=59 for normal tissue, n=515 for lung adenocarcinoma tissue, p<0.001) and lung squamous cell carcinoma (n=52 for normal tissue, n=503 for lung squamous cell carcinoma tissue, p<0.001) in TCGA datasets. (D) Western Blot analysis of Sirt3 expression in bronchial epithelial cells (BEAS), and different lung cancer cells (A549, H1299, H460). (E) Western Blot analysis of Sirt3 expression in bronchial epithelial cells and different lung cancers after 8Gy irradiation at different time points.

Firstly, we performed a tissues microarray by using patients samples and found that the expression level of Sirt3 in lung cancer tissues was considerably higher than that in para-cancerous tissues (Fig. 1A, B). The TCGA database showed the same trend, in lung adenocarcinoma and lung squamous cell carcinoma, the expression level of Sirt3 in primary tumor was significantly higher than that in normal lung tissue adjacent to cancer (Fig. 1C). Next, we examined the basal expression level of Sirt3 in human bronchial epithelial cells (BEAS-2B), various human lung cancer cells (A549, H460, H1299) by Western Blotting assay. The results showed that the expression level of Sirt3 in lung cancer cells was much higher than in normal bronchial epithelial cells (Fig. 1D). We explored whether irradiation has an impact on Sirt3 expression in human bronchial epithelial cell and lung cancer cells. The results showed that after 8Gy irradiation, the expression level of Sirt3 in BEAS-2B cell increased markedly at 8 hours after irradiation, while in lung cancer cells A549 and H1299, the Sirt3 expression level was significantly decreased at 12 hours after irradiation (Fig. 1E). These data showed that upregulation of Sirt3 in lung cancer might be a critical factor responsive to ionizing radiation.

### Sirt3 affects the radiosensitivity in lung cancer cells

**Figure 2:**
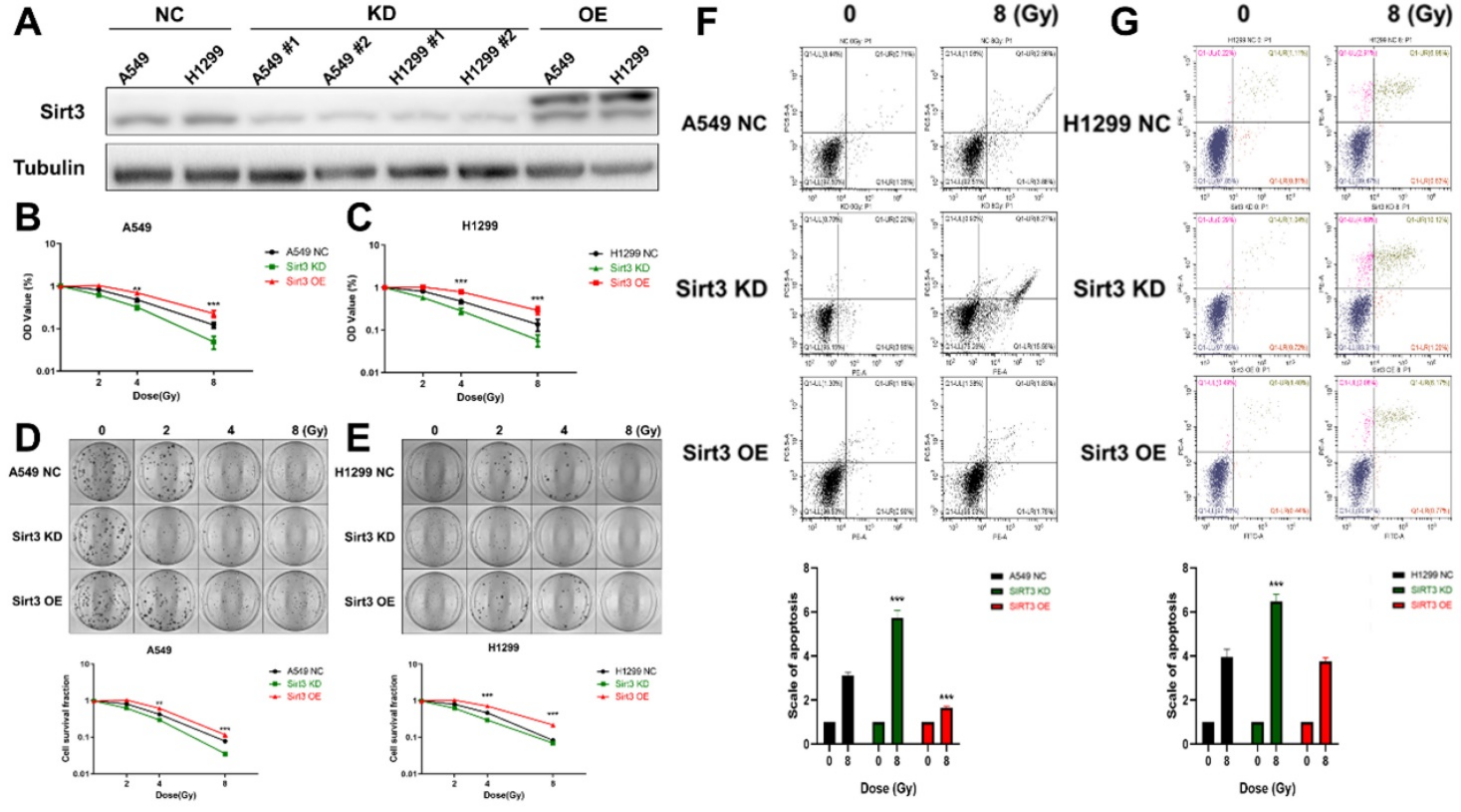
** Sirt3 affects the radiosensitivity in lung cancer cells.** (A) The level of Sirt3 expression shown by Western Blot analysis in A549 and H1299 infected with Sirt3-specific shRNA and overexpression lentivirus. (B and C) Cell viability of A549 and H1299 in different groups after 0, 2, 4, 8 Gy irradiation determined by CCK-8 assay. (D and E) Cell survival and its representative images of A549 and H1299 in different groups after 0, 2, 4, 8 Gy irradiation was determined by colony formation. (F and G) The apoptosis of A549 and H1299 after 8Gy irradiation at 24h in different groups was analyzed by flow cytometry. Values are given as mean±SEM, n = 6 for each group per time point, **P<0.01 and ***P<0.001 versus negative control shRNA group.

To investigate the effect of Sirt3 on the radiation sensitivity of lung cancer cells, we constructed Sirt3 knockdown shRNAs and overexpression plasmid, and further transfected it to A549 and H1299 cells by lentivirus infection, then verified their knockdown and overexpression efficiency by Western Blot (Fig. 2A). Next, we examined cell viability of these cell lines by CCK-8, and found that Sirt3 knockdown cell was more sensitive to irradiation and show lower cell viability after irradiation (Fig. 2B, C). At the same time, it was found that Sirt3 knockdown cell formed fewer colonies after irradiation than NC group, further indicated that it is more sensitive to irradiation, while Sirt3 overexpression cell was not significantly different from the NC group (Fig. 2D, E). By using flow cytometry assay, we found that after 8Gy irradiation, the proportion of apoptotic cells in Sirt3 knockdown group was significantly higher than that in NC group, while the percentage of apoptotic cells in Sirt3 overexpression group was significantly decreased (Fig. 2F, G).

### Sirt3 promote radiation-induced DNA damage repair and cell cycle arrest

**Figure 3:**
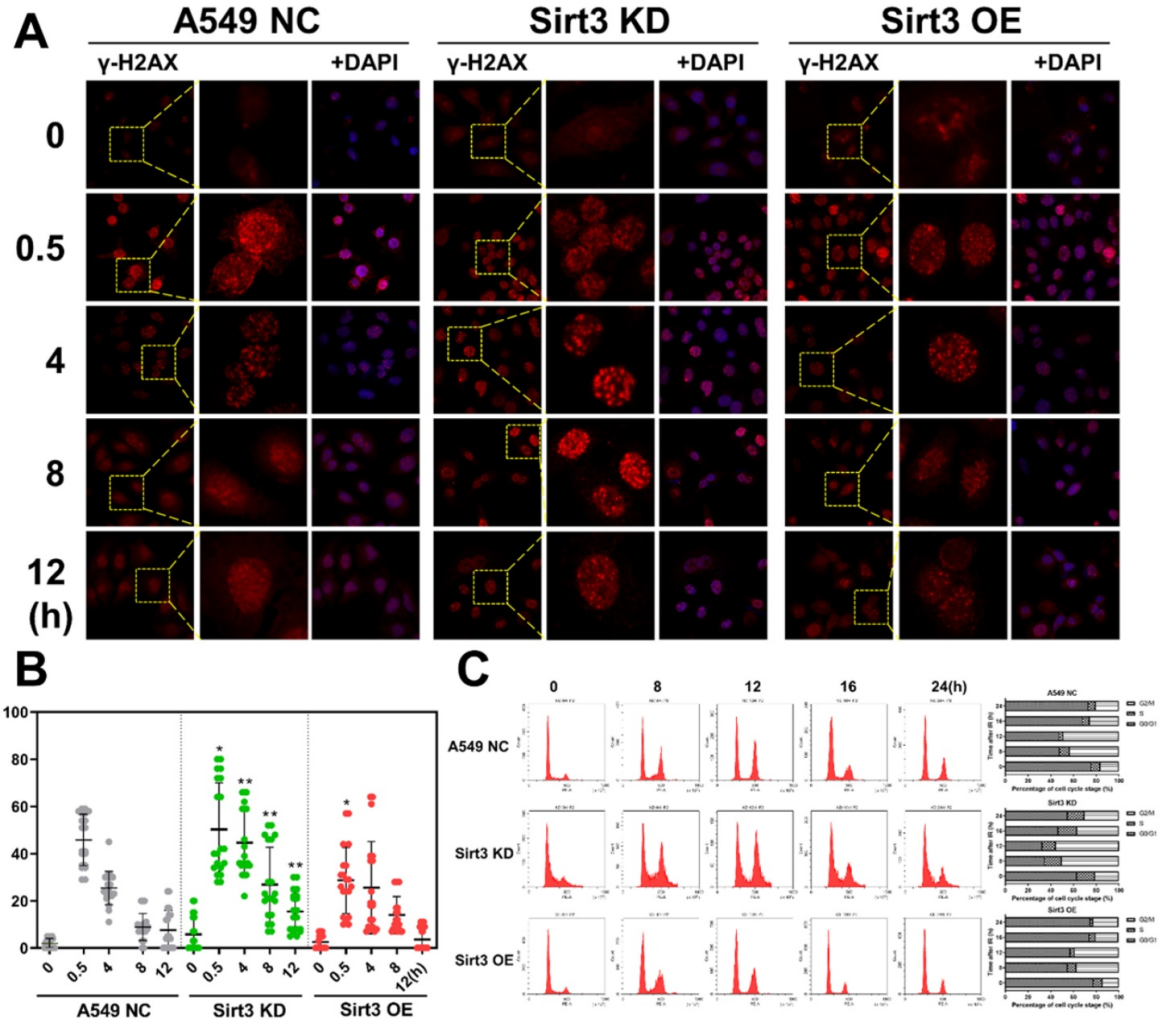
** Sirt3 affects the process of radiation-induced DNA damage repair and cell cycle arrest.** (A) The γ-H2AX foci in A549 after 4Gy irradiation in different groups was analyzed by immunofluorescence staining. (B) Quantitative analysis of the number of γ-H2AX foci per cell in different groups, n = 30 for each group per time point. (C) The cell cycle of A549 after 8Gy irradiation at 48h in different groups was analyzed by flow cytometry, n = 6 for each group per time point. Values are given as mean±SEM. *P<0.05 and **P<0.01 versus negative control shRNA group.

As to DNA damages, Sirt3 knockdown group showed more γ-H2AX foci remained unrepaired than the NC group, and more foci were present until 12 hours after irradiation, indicated that A549 cell with Sirt3 knockdown had more severe DSBs and slower DSBs repair after 4Gy irradiation. While results in the Sirt3 overexpression group were reversed (Fig. 3A, B). Cells are largely arrested in the G2/M phase to repair the DNA damage caused by irradiation. In this study, we found that in NC group, 8Gy irradiation caused G2/M cell cycle arrest of A549, and it was most severe in 12 hours after irradiation, then gradually recovered. In the Sirt3 knockdown group, the G2/M phase arrest caused by irradiation was more severe and the recovery was slower, indicated that the A549 cells in this group had more profound DNA damage and less repair efficiency. In contrast, the G2/M phase arrest in Sirt3 overexpression group was reduced and recovered faster than NC group (Fig. 3C).

### Sirt3 is required for the activation of ATM-Chk2 mediated DNA repair

**Figure 4:**
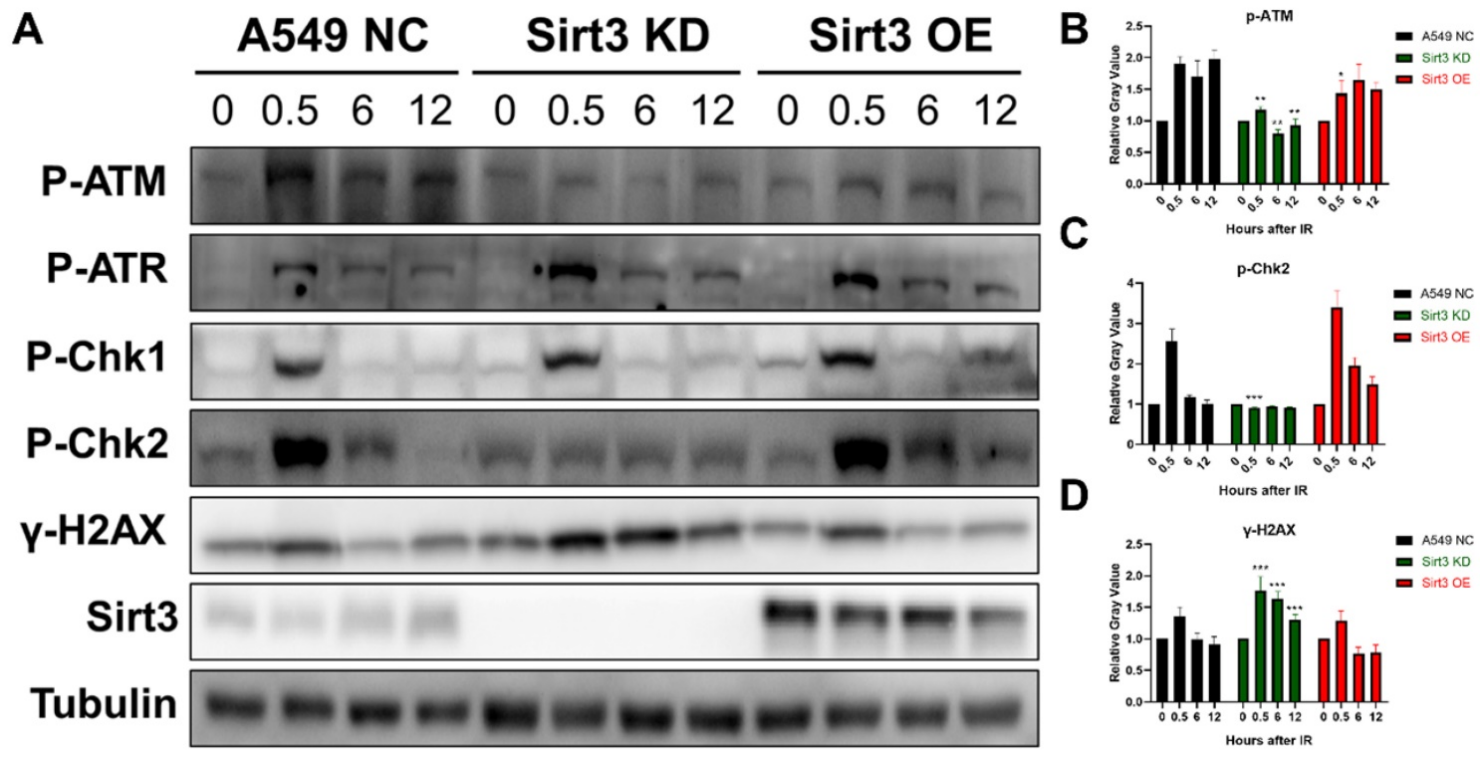
** Sirt3 is required for the activation of ATM-Chk2 mediated DNA repair.** (A) Western Blot analysis of the protein expression in DNA Damage Response pathway in A549 after 8Gy irradiation at different time points. (B) Gray-scale analysis of Western Blot analysis results, n = 6 for each group per time point. Values are given as mean±SEM. *P<0.05, **P<0.01 and ***P<0.001 versus negative control shRNA group.

To explore the role of Sirt3 in the signaling pathway in DNA damage repair (DDR), proteins of each group at 0, 0.5, 6, and 12 hours were extracted after 8Gy irradiation. The results showed that in NC group, activation of ATR-Chk1 and ATM-Chk2 pathway occurred after irradiation, and the activation gradually diminished up to 24h. The phosphorylation of ATR and Chk1 in the Sirt3 knockdown group was consistent with NC group, while the activation of p-ATM and p-Chk2 was significantly inhibited. As an indicator of DNA damage, γ-H2AX level remains upregulated at later time points, which was consistent with the immunofluorescence results (Fig. 4). These findings indicated that Sirt3 is required for the activation of ATM-Chk2 signaling pathway.

### Sirt3 confers radioresistance in in situ murine lung cancer model

**Figure 5:**
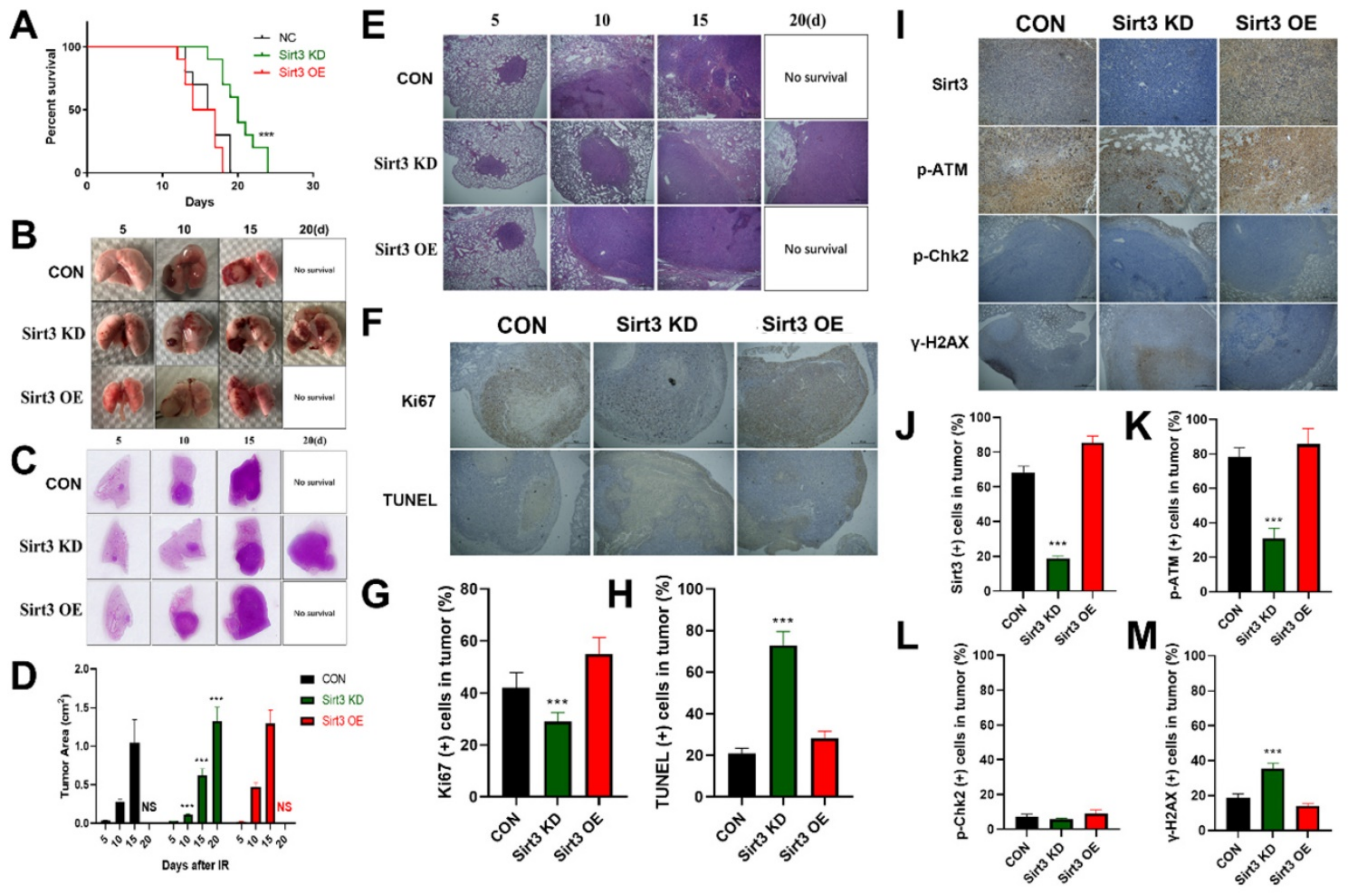
** Sirt3 confers radioresistance in in situ murine lung cancer model.** (A) The animal survival of lung tumor-bearing mouse model in different groups after 15Gy local irradiation in chest. (B) Representative images of lung tissue in different groups from 5 to 20 days after 15Gy local irradiation in chest. (C and E) Representative 1× and 40× images of HE immunohistochemical staining of lung tissue sections in different groups at 5 to 20 days after 15Gy local irradiation in chest. (D) Quantitative analysis of the ratio of tumor area to lung tissue in HE immunohistochemical staining. (F) Representative 40× images of Ki67 and TUNEL immunohistochemical staining of lung tissue sections in different groups at 10 days after 15Gy local irradiation in chest. (G and H) Representative the percentage of Ki67 (+) and TUNEL (+) cells in tumor by quantitative analysis. (I) Representative 100× images of Sirt3, p-ATM, p-Chk2, and γ-H2AX immunohistochemical staining of lung tissue sections in different groups at 24 hours after 15Gy local irradiation in chest. (J, K, L and M) Representative the percentage of Sirt3 (+), p-ATM (+), p-Chk2 (+), and γ-H2AX (+) cells in tumor by quantitative analysis. Values are given as mean±SEM, n = 6 for each group per time point, **P<0.01 and ***P<0.001 versus negative control shRNA group.

In order to explore the in vivo effect of Sirt3 on lung cancer, we constructed a mouse lung cancer radiotherapy model, and implanted Sirt3 NC, Sirt3 knockdown, and overexpression Lewis lung cancer (LLC) cells in the left lower lung lobe of C57BL/6 mice. One week later, the mice were subjected to single localized γ-irradiation at a dose of 15 Gy on lung area. Survival results showed that the survival time of the Control and Sirt3 overexpression groups was less than 20 days, while the knockdown group had the longest survival time till 24 days, showed a significant survival advantage (Fig. 5A). General view of lung tissues showed that compared with the Control group, the lung tumor in Sirt3 knockdown group grew slower, and had more profound hemorrhagic necrosis (Fig. 5B). In HE staining, we found the same trend, indicated that Sirt3 knockdown tumor cell was more severely damaged by irradiation at the same dose(Fig. 5C-E). TUNEL staining can better reflect the degree of radiation-induced apoptosis of tumor cells, and Ki67 staining reflects the proliferation ability of tumor cells. Next, we found that in the Sirt3 knockdown group, radiation-induced apoptosis of tumor cells was more extensive and severe, reflected its better radiation sensitivity. At the same time, the proliferation ability of tumor cells in Sirt3 knockdown group was significantly weaker than that in Control group and Sirt3 OE group, indicated that the inhibit ability of radiation in this group was much stronger(Fig. 5F-H). By immunohistochemical staining of Sirt3, p-ATM, p-Chk2 and γ-H2AX in each group of tumor tissues, it was also found that the activation of γ-H2AX was more pronounced in Sirt3 knockdown group, and the radiation-induced activation of p-ATM was significantly attenuated. However, in p-Chk2 immunofluorescence staining, compared to normal lung tissues, we find few p-Chk2 positive staining cells in tumor tissues (Fig. 5I-M).

## Discussion

To our knowledge, this is the first study demonstrating the role of Sirt3 in radiosensitivity of lung cancer cells through regulating ATM mediated DNA damage repair. In this study, we demonstrated that Sirt3 is highly expressed in NSCLC cell lines, tissues, as well as patients data derived from TCGA database. As a radiation responsive gene, Sirt3 also participate in the radiation resistance of lung cancer cells. Our data also showed that Sirt3 deficiency results in more cells arrested in G2/M phase. Sirt3 is also required for the activation of ATM-Chk2 pathway, instead of DNA-PKcs or ATR. Finally, using our in situ lung cancer model, we found that Sirt3 confers the radioresistance of lung cancer in vivo.

Radiotherapy is an indispensable strategy for NSCLC when used alone or combined with chemotherapy as well as targeted therapy^11^,^12^. When some patients benefit from this technique, a large proportion are characterized with a radioresistant property 13-15. Even for those sensitive, radioresistance gradually occurs as radiotherapy applied^16^. So scientists tried to identify novel radiosentizing target to improve the therapy of lung cancer. Sirtuins are a family of silent information regulator (Sirtuins) which was firstly identified in aging the cellular senescence 17,18. Among all, Sirt1, Sirt6 and Sirt7 was demonstrated to involve in DNA damage repair, the over-activation of which accounts mainly for radioresistance of cancer 19,20. In the present study, we demonstrated that Sirt3 was also critical for the activation of ATM-Chk2 signaling pathway. However, the activation of ATR or chk1 remains unchanged. These data indicated that Sirt3 might regulates ATM mediated HR repair. Moreover, we also found that Sirt3 deficiency caused impairment in DNA damage repair in terms of γ-H2AX foci. These findings provide novel mechanism of radioresistance in NSCLC.

Recently, Sirt3 was found to be involved in cancer development and progression 8,21. And Sirt3 is also critical for mitochondrial function, DNA damage, cell metabolism as well as cell apoptosis 5,22. Up to now, Sirt3 has been studied in many cancer types, including esophageal cancer^23^, oral cancer^24^, colon cancer^25^, breast cancer^26^ *etc*. However, whether Sirt3 plays a role as oncogene or tumor suppressor is still controversial 27. For lung cancer, is has been reported that Sirt3 is related to the poor prognostic 28. However, the role of Sirt3 in radiotherapy of lung cancer is unclear. Cellular radiosensitivity is the key basis of tumor radiation response. Then we determined the cellular response of NSCLC to ionizing radiation when Sirt3 is overexpressed or downregulated. In our present study, we found that cells with Sirt3 knockdown was sensitive to ionizing radiation, while Sirt3 overexpression increased the radioresistance of NSCLC cells. Knockdown of Sirt3 also enhanced radiation-induced apoptosis and G2/M cell cycle arrest. These data suggested that Sirt3 is related to a radioresistant phenotype, which provide a potential target for radiosensitization.

In vivo tumor model is critical for the evaluation of tumor progression and therapy. For lung cancer radiotherapy, several animal models has been developed for the study of cancer response according to previous studies 29,30. For instance, subcutaneous tumor bearing model is widely used when for test of drugs or radiotherapy 31. Another model is Kras-induced lung cancer, in which mice was generated with a Kras mutant 30. However, neither the subcutaneous model nor Kras mutant model can reflect the real environment in lung cancer. In our present study, we generated an in situ lung cancer model, and we injected the Sirt3 modified cells directly into the lung tissues. After then, radiotherapy was applied. It was found that Sirt3 deficiency increased the radiosensitivity in vivo, and targeting Sirt3 also reduced the activation of DNA damage signaling pathway. These findings provide evidence that Sirt3 is potential therapeutic target in terms of animal model. However, we did not find p-Chk2 positive staining cells in tumor tissues, this phenomenon may be due to a significant decrease of phosphorylation Chk2 in tumor cells 10 days after irradiation, or the expression level of p-Chk2 is too low in LLC cells, the specific cause needs to be identified in further experiments.

## Conclusions

In conclusion, we demonstrated that Sirt3 is upregulated in NSCLC tissues and cell lines. As a radiation responsive gene, Sirt3 confers radioresistance in NSCLC cell lines in terms of survival assay and apoptosis assay. DNA damage repair impairment and cell cycle arrest was also observed in Sirt3 knockdown cells upon irradiation. Our findings provide novel mechanism of radiation resistance and suggests Sirt3 as a potential target of radiosensitization in NSCLC.

## Abbreviations

NSCLC: non-small cell lung cancer
Sirt3: Sirtuin 3
LLC: Lewis lung cancer
CCK-8: Cell Counting Kit-8
PE: plating efficiency
TUNEL: terminal transferase-mediated dUTP nick end labeling
TCGA: The Cancer Genome Atlas
DDR: DNA damage repair
ATR: Ataxia Telangiectasia And Rad3-Related Protein
ATM: Ataxia Telangiectasia Mutated
Chk1: Checkpoint Kinase 1
Chk2: Checkpoint Kinase 2
Ki67: Marker Of Proliferation Ki-67
DNA-PKcs: DNA-Dependent Protein Kinase Catalytic Subunit
HR: Homologous recombination
